# Trends and disparities in urinary tract infections-related mortality in the United States from 1999 to 2023: Insights from CDC WONDER

**DOI:** 10.1097/MD.0000000000049032

**Published:** 2026-05-22

**Authors:** Shehdev Meghwar, Riya Bhagwan, Sayed Maisum Mehdi Naqvi, Fnu Urooba, Chris Jerical, S. M. Aleem Hussain, Aseef Rehman, Madina Memon, Vishan Das, Mohammed Hammad Jaber Amin, Shree Rath, Sabahat Ul Ain Munir Abbasi

**Affiliations:** a Department of Medicine, Liaquat University of Medical and Health Sciences, Jamshoro, Pakistan; b Department of Medicine, Dow University of Health Sciences, Karachi, Pakistan; c Department of Medicine, Dow International Medical College, DUHS, Karachi, Pakistan; d Department of Medicine, CMH Institute of Medical Science, Multan, Pakistan; e Department of Medicine, Services Institute of Medical Sciences, Lahore, Pakistan; f Department of Medicine, University College of Medicine and Dentistry, Lahore, Pakistan; g Faculty of Medicine, Alzaiem Alazhari University, Khartoum, Sudan; h Department of Medicine, All India Institute of Medical Sciences, Bhubaneswar, India; i Department of Medicine, Allama Iqbal Medical College, Jinnah Hospital, Lahore, Pakistan.

**Keywords:** disparities, mortality, United States, urinary tract infections

## Abstract

Urinary tract infections (UTIs) rank among the most common bacterial infections globally, impacting around 404 million individuals each year. This research investigates trends in mortality associated with UTIs in adult patients across the United States from 1999 to 2023. We retrospectively examined death statistics from the CDC WONDER database, examining mortality trends where UTI was the primary or contributing cause. Age-adjusted mortality rates (AAMR) per 1,00,000 were computed and categorized by sex, race/ethnicity, urbanization, state, and census region. Trends were evaluated using joinpoint regression to determine annual percentage change (APC) with 95% confidence intervals. From 1999 to 2023, there were a total of 10,83,913 UTI-related deaths among individuals aged 25 and older. The overall AAMR decreased from 1999 to 2018 (APC: −0.80; 95% CI: −1.13 to −0.46), followed by a significant increase up to 2023 (APC: 3.95; 95% CI: 1.60–6.36). Females consistently displayed higher AAMRs compared to males. Non-Hispanic (NH) American Indian/Alaskan Native individuals experienced the highest (AAMR) among racial groups. In terms of geography, nonmetropolitan areas exhibited higher rates than metropolitan regions, while the South reported the highest AAMR when considering census regions. Tennessee had the highest AAMR (30.48) between 1999 and 2020. Mortality related to UTIs has been on the rise since 2018, with the most notable rates found among females, NH American Indian/Alaskan Native, nonmetropolitan residents, and those in the Southern region. These disparities emphasize the necessity for ongoing initiatives and targeted interventions aimed at these high-risk groups.

## 1. Introduction

A urinary tract infection (UTI) is a broad diagnostic term that describes infections located throughout the urinary system and related tissues, such as the urethra, prostate, bladder, or kidneys.^[1]^ They affect approximately 404 million people worldwide and nearly 9.8 million individuals annually in the United States.^[2,3]^ Although most UTIs are uncomplicated and respond to antibiotic therapy, complications such as pyelonephritis, sepsis, and systemic involvement can occur and are associated with substantial mortality, particularly among older and immunocompromised patients and those with comorbidities.^[4]^ Recent studies have also reported an increasing burden of UTI-related hospitalizations and mortality.^[5]^

Advances in diagnostics, antibiotic therapies, and infection control have improved clinical care, yet the long-term patterns of UTI-related mortality in the United States remain incompletely characterized. In particular, disparities by race/ethnicity, age, and geographic region may drive heterogeneous outcomes across populations.^[6,7]^ UTIs also impose a considerable economic burden, with billions spent annually in the United States on hospitalizations, emergency visits, and antibiotic therapy.^[8]^ The growing challenge of antimicrobial resistance (AMR) among urinary pathogens further complicates management, which leads to prolonged hospitalization, recurrent infections, and an increased mortality rate. There are also several age-related factors, such as urinary retention, catheter use, and many chronic illnesses, which significantly contribute to the high incidence of UTIs and risk of adverse outcomes in the aging population.^[9]^ Overall, AMR and population aging have increased the health impact of UTIs, emphasizing the need for continuous monitoring and effective antimicrobial stewardship strategies. Women also account for the majority of cases due to anatomical susceptibility.^[10]^

Despite these concerns, existing national data have not fully described long-term trends in UTI-related mortality nor quantified how these trends differ across key demographic and geographic subgroups. We analyzed US mortality records from 1999 to 2023 in a nationwide retrospective analysis using the CDC WONDER database. The analysis aimed to quantify temporal trends in deaths linked to UTIs and to evaluate disparities across age, sex, race/ethnicity, and geographic regions, thereby providing evidence to prioritize public-health and clinical interventions for populations at greatest risk. We hypothesized that mortality rates associated with urinary tract infections have undergone notable changes over time, and that these trends vary among important demographic groups (age, sex, race/ethnicity) and geographical areas in the United States.

## 2. Materials and methods

### 2.1. Patient eligibility and screening

The CDC WONDER (Wide-ranging Online Data for Epidemiologic Research) database served as the source for mortality statistics related to deaths from UTIs in the United States.^[11]^ This database gathers mortality information derived from death certificate records across all 50 states and the District of Columbia. To analyze mortality trends, the multiple cause-of-death public use records were utilized to identify instances where UTIs were documented as either a contributing or underlying cause of death. The identification of UTI cases was conducted using codes from the International Classification of Diseases 10th Revision Clinical Modification (ICD-10-CM),^[12]^ specifically N10, N12, N13.6, N15.1, N30, N34, N39.0, O23, and T83.5, for individuals aged 25 years and older. The data assessed were gathered from 1999 to 2023. These codes have been used in previous studies to measure the UTI-related burden.^[13]^

No institutional review board approval was necessary for this study, as the analysis was based on de-identified disease surveillance data obtained from the CDC databases and complied with STROBE (Strengthening the Reporting of Observational Studies in Epidemiology) guidelines.^[14]^

### 2.2. Data extraction

Data concerning fatalities in patients with UTIs, including population sizes, years, demographics such as age, gender, and ethnicity or racial background, as well as age groups, census regions, urbanization classifications, states, and locations of death, were gathered. Ethnic groups were classified into Hispanic (Latino) and non-Hispanic (NH), with further breakdown into White, Black or African American, American Indian or Alaska Native, and Asian or Pacific Islanders. Age categories were analyzed in 10-year increments, starting from 25 years and continuing to those aged 85 and older. The US Census Bureau divides the country into 4 principal regions: the Midwest, West, Northeast, and South. The classification of urban and rural populations followed the National Center for Health Statistics Urban-Rural Classification Scheme, which is based on the 2013 US Census data. The analyses stratified by urbanization were restricted to data available up until 2020, as the CDC WONDER database does not offer county-level urbanization classifications for years following this. Death locations encompassed medical facilities (including inpatient, outpatient, deaths on arrival, and unknown medical facility), decedent’s home, hospice facility, nursing home/long-term care, and other.

### 2.3. Data synthesis

Age-adjusted mortality rates (AAMRs) and crude mortality rates (CMRs) for UTIs, expressed per 1,00,000 individuals according to the 2000 US population, were computed according to CDC guidelines. Factors such as year, census region, state, race/ethnicity, and metropolitan classification were utilized to analyze the demographic and regional data obtained from the CDC WONDER database, along with the 95% confidence intervals (CIs) for the AAMR.

Temporal trends in AAMR were evaluated using the Joinpoint Regression Program version 5.4.0, which fits a sequence of log-linear models allowing the analyst to specify a minimum and maximum number of joinpoints (i.e., points at which the trend slope changes).^[15]^ The software begins with the minimum (often 0 joinpoints) and tests whether adding additional joinpoints up to the specified maximum improves model fit, using a Monte Carlo permutation method to assess the significance of each change. Model selection is generally based on the permutation test and often uses criteria such as the Bayesian information criterion (BIC) or modified goodness-of-fit tests to choose the simplest model that adequately describes the data. For each trend, APCs and CIs were calculated. A sequential approach to hypothesis testing was employed to determine the best joinpoint model. The analysis started with a model that had no joinpoints. Iteratively, more joinpoints were introduced, and at each stage, the more complex model was assessed against the simpler one using the Monte Carlo permutation test. The mortality trend’s slope was significantly different from zero if there was a notable rise or decline in the APCs, with statistical significance established at *P* < .05 utilizing 2-tailed *t* tests. The APCs, average annual percent change (AAPCs), and 95% CIs encapsulated the mortality trend from 1999 to 2023.

## 3. Results

A total of 10,83,913 deaths were attributed to UTI-related mortality among adults (aged 25 and above) in the United States from 1999 to 2023. Out of which 6,87,067 deaths were recorded in females, and 3,96,846 deaths were recorded in males (Table S1).

Information on deaths across care settings revealed that the highest mortality occurred in medical facilities, including inpatient, outpatient, and dead-on-arrival cases (62.25%). This was followed by deaths in nursing homes or long-term care facilities (21.53%), decedents’ homes (9.10%), and hospice settings (4.68%; Table S2).

### 3.1. Annual trends for UTI-related mortality

The AAMR for UTI-related deaths exhibited a distinct biphasic pattern from 1999 to 2023, characterized by an initial decline followed by a subsequent increase.

**Figure 1.**
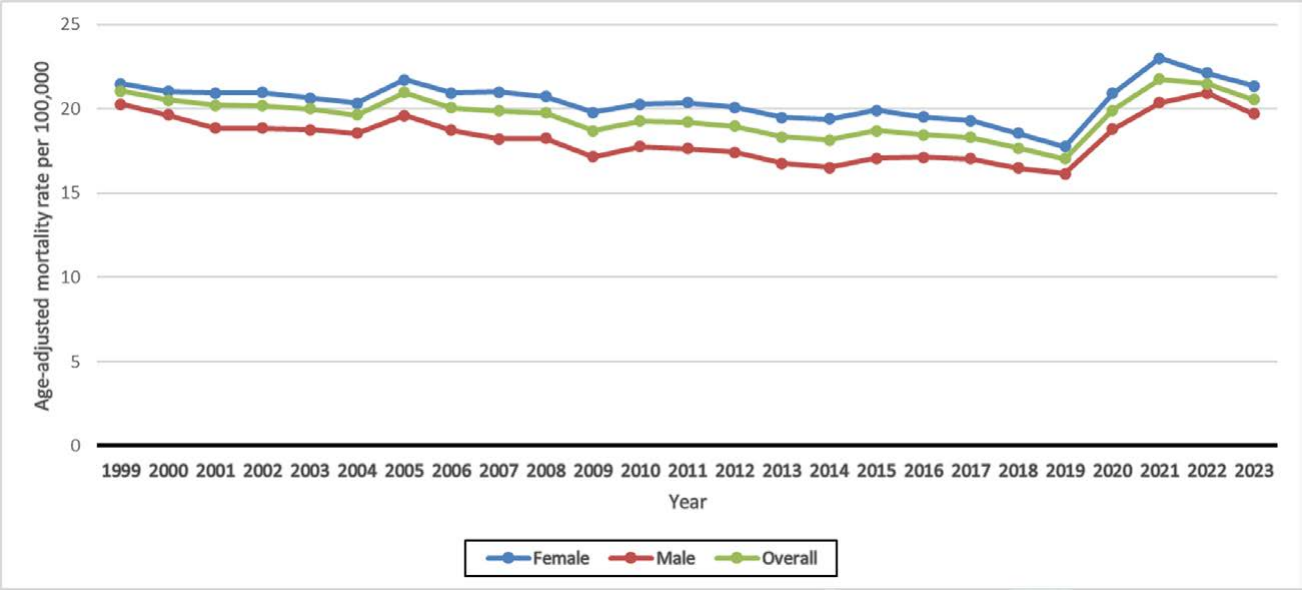
Age-adjusted mortality rates for UTI-related deaths, stratified by sex. UTI = urinary tract infections.

The overall AAMR was recorded as 21.04 in 1999 and 20.5 in 2023 (AAPC: 0.17; 95% CI: −0.34 to 0.69; *P* = .51). During the initial segment from 1999 to 2018, a decline was observed from 21.08 to 17.66 (APC: −0.80; 95% CI: −1.13 to −0.46; *P* < .001), followed by a sharp upward trend, with a significant increase to 20.55 in 2023 (APC: 3.95; 95% CI: 1.60–6.36; *P* = .002; Fig. 1 and Table S3).

### 3.2. UTI-related mortality trends stratified by gender

While stratifying data by gender, females exhibited consistently higher AAMRs compared to males throughout the study period. The female-specific AAMR was recorded 21.46 in 1999 and 21.32 in 2023 (AAPC: 0.26; 95% CI: −0.13 to 0.65; *P* = .195). In contrast, the male-specific AAMR was observed at 20.25 in 1999 and 19.66 in 2023. (AAPC: 0.30; 95% CI: −0.22 to 0.82; *P* = .26).

Among females, the AAMR showed an initial slight decrease from 21.46 in 1999 to 17.75 in 2019 (APC: −0.56; 95% CI: −0.77 to −0.34; *P* < .001), followed by a sharp upward trend until 2023 (APC: 4.44; 95% CI: 2.13–6.80; *P* < .001), reaching the AAMR of 21.32.

Similarly, among males, the AAMR showed an initial decline from 20.25 in 1999 to 16.47 in 2018 (APC: −0.92; 95% CI: −1.28 to −0.57; *P* < .001), followed by a sharp increasing trend until 2023 (APC: 5.09; 95% CI: 2.72–7.50; *P* < .001), reaching the AAMR of 19.66 (Fig. 1 and Table S4).

### 3.3. UTI-related mortality trends stratified by race

Over the past 2 decades, the highest recorded AAMRs were observed among NH American Indian or Alaskan Native individuals (AAPC: 0.97; 95% CI: −1.12 to 3.10; *P* = .365) and NH Black or African American individuals (AAPC: −1.46; 95% CI: −2.26 to −0.66; *P* < .001). In contrast, the lowest rates were recorded among Asian or Pacific Islander individuals (AAPC: −0.33; 95% CI: −1.14 to 0.48; *P* = .424).

Among NH American Indian or Alaska Native individuals, the AAMR initially rose from 18.54 in 1999 to 26.63 in 2011 (APC: 1.99; 95% CI: 0.74–3.26; *P* = .004), followed by a decline to 21.42 in 2018 (APC: −3.04; 95% CI: −5.64 to −0.37; *P* = .029). It then started rising again until 2021 (APC: 13.26; 95% CI: −1.61 to 30.38; *P* = .079), increasing significantly to 30.50 in 2021. From 2021 onwards, a decrease in trend was observed again, having an AAMR value of 25.17 in 2023 (APC: −7.76; 95% CI: −18.81 to 4.79; *P* = .196).

Among NH Black or African American individuals, an initial decrease in AAMR was observed from 31.92 in 1999 to 19.57 in 2018 (APC: −2.70; 95% CI: −2.88 to −2.52; *P* < .001), followed by a significant increase until 2021 (APC: 8.44; 95% CI: 2.28–14.98; *P* = .009), reaching an AAMR of 24.32. It then started declining again, having an AAMR of 22.27 in 2023 (APC: −3.71; 95% CI: −8.80 to 1.61; *P* = .16).

NH White individuals showed an initial decrease in AAMR from 20.34 in 1999 to 18.33 in 2018 (APC: −0.41; 95% CI: −0.74 to −0.08; *P* = .017), followed by a notable increase until 2023, reaching an AAMR of 21.59 (APC: 4.27; 95% CI: 1.93–6.67; *P* = .001).

For Hispanic or Latino individuals, AAMR declined from 17.55 in 1999 to 13.16 in 2018 (APC: −1.32; 95% CI: −1.84 to −0.80; *P* < .001). It then showed an upward trend until the end of the study period (APC: 4.26; 95% CI: 1.29–7.32).

**Figure 2.**
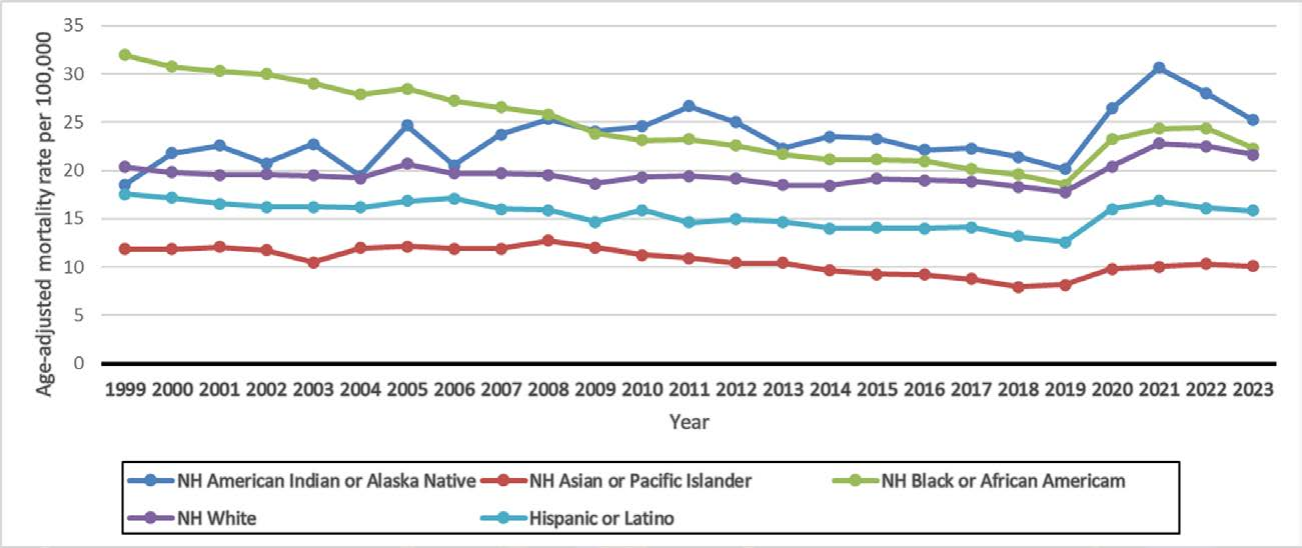
Age-adjusted mortality rates for UTI-related deaths, stratified by race. NH = non-Hispanic, UTI = urinary tract infections.

NH Asian or Pacific Islander individuals showed a slight increase in AAMR from 11.79 in 1999 to 12.75 in 2008 (APC: 0.76; 95% CI: −0.73 to 2.28; *P* = .30), followed by a notable drop to 7.94 in 2018 (APC: −3.82; 95% CI: −4.91 to −2.72; *P* < .001). It then started rising again until 2023 (APC: 4.94; 95% CI: 2.54–7.39; *P* < .001), reaching an AAMR of 10.06 (Fig. 2 and Table S5).

### 3.4. UTI-related mortality trends stratified by geography

A substantial variation in AAMR was observed across US states from 1990 to 2020. The highest AAMR was recorded in Tennessee, at 30.48, while the lowest was observed in Hawaii, at 11.15. States within the upper 90th percentile for AAMR included Tennessee, Oklahoma, Mississippi, West Virginia, South Carolina, and Kentucky, reflecting elevated mortality burdens. In contrast, states within the lower 10th percentile, Hawaii, Florida, Arizona, Nevada, Minnesota, and New Jersey, exhibited comparatively reduced mortality burdens (Table S6).

Whereas; from 2021 to 2023 Oklahoma observed the highest mortality rate (AAMR: 51.51), while the lowest rate was observed in Hawaii (AAMR: 10.17). Kentucky, South Dakota, West Virginia, and Wyoming were also included in top 90th percentile (Table S6).

In terms of urbanization, nonmetropolitan areas exhibited a stable trend in AAMR, which remained consistently higher than that in metropolitan areas throughout the 24-year study period.

**Figure 3.**
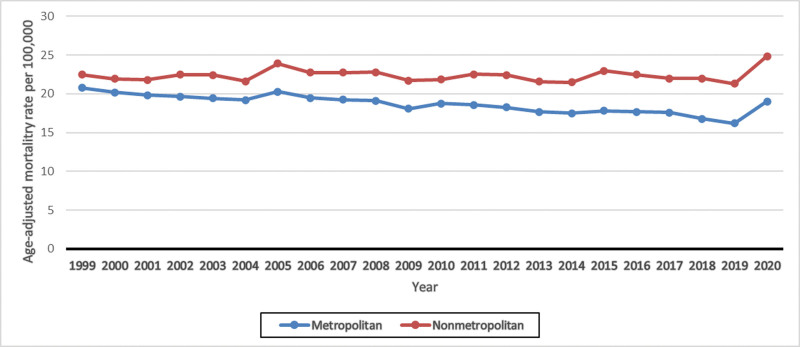
Age-adjusted mortality rates for UTI-related deaths, stratified by urbanization. UTI = urinary tract infections.

In nonmetropolitan areas, the AAMR was recorded 22.42 in 1999 and 24.78 in 2020 (AAPC: 0.06; 95% CI: 0.21–0.32; *P* = .66). In contrast, in metropolitan areas, the AAMR decreased significantly from 20.74 in 1999 to 18.98 in 2020 (AAPC: −0.82; 95% CI: −1.06 to −0.56; *P* = .00001; Fig. 3 and Table S7).

### 3.5. UTI-related mortality trends stratified by census region

Mortality trends also showed disparities across US Census regions during the last 24 years, from 1999 to 2023. The highest AAMRs were observed in the South region (AAPC: −0.09; 95% CI: −1.10 to 0.94; *P* = .87), and the lowest AAMRs were recorded in the Northeast region (AAPC: −0.03; 95% CI: −0.56 to 0.50; *P* = .90).

In the Northeast region, the AAMR decreased from 17.91 in 1999 to 13.94 in 2019 (APC: −0.90; 95% CI: −1.19 to −0.62; *P* < .001), followed by an increase to 17.15 until 2023 (APC: 4.44; 95% CI: 1.31–7.66; *P* = .007).

In the Midwest region, the AAMR decreased from 19.88 in 1999 to 16.82 in 2018 (APC: −0.76; 95% CI: −1.05 to −0.47; *P* < .001). And, from 2018 onward, a nonsignificant sharp rise was observed in AAMR until 2021 (APC: 6.66; 95% CI: −2.73 to 16.95; *P* = .16) before it declined (APC: 3.20; 95% CI: −11.30 to 5.63; *P* = .44).

In the South region, the AAMR decreased from 23.97 in 1999 to 19.50 in 2018 (APC: −1.05; 95% CI: −1.30 to −0.81; *P* < .001), followed by an increase to 24.65 in 2021 (APC: 8.10; 95% CI: 0.42–16.36; *P* = .04). Then a nonsignificant decrease to 22.97 occurred in 2023 (APC: −2.61; 95% CI: −9.04 to 4.26; *P* = .42).

**Figure 4.**
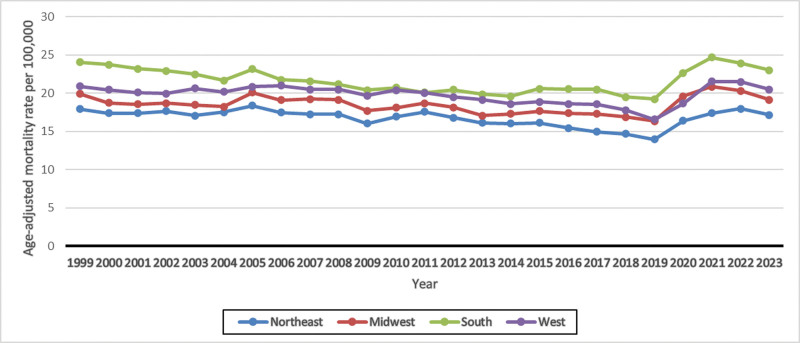
Age-adjusted mortality rates for UTI-related deaths, stratified by census region. UTI = urinary tract infections.

In the West region, a decline in AAMR was observed from 20.84 in 1999 to 16.55 in 2019 (APC: −0.78; 95% CI: −1.08 to −0.49; *P* < .001). This was followed by a moderate increase to 20.42 in 2023 (APC: 4.52; 95% CI: 1.54–7.59; *P* = .005; Fig. 4 and Table S8).

### 3.6. UTI-related mortality trends stratified by age group

CMRs revealed distinct patterns across various age groups from 1999 to 2023. The highest CMR was recorded in older adults aged 85 + years (AAPC: −0.54; 95% CI: −0.94 to −0.13; *P* = .01).

In the 25 to 34 years cohort, a gradual rise in CMR occurred from 1999 to 2018, with the rate increasing from 0.30 to 0.36 (APC: 1.42; 95% CI: 0.62–2.22; *P* = .001). This was followed by a sharper escalation between 2018 and 2023 (APC: 8.35; 95% CI: 3.14–13.81; *P* = .003), with the crude rate reaching 0.52 by 2023.

In the 35 to 44 years cohort, a modest rise in CMR occurred from 1999 to 2018, with the rate increasing from 0.82 to 0.95 (APC: 0.79; 95% CI: 0.46–1.12; *P* < .001). This was followed by a sharp increase to 1.41 in 2021 (APC: 15.21; 95% CI: 4.06–27.54; *P* = .009). From 2021 to 2023, the trend showed a nonsignificant decline to 1.29 in 2023 (APC: −2.62; 95% CI: −10.68 to 6.18; *P* = .53).

In the 45 to 54 years cohort, a steady rise occurred from 1999 to 2018, with the rate increasing from 2.14 to 2.75 (APC: 1.33; 95% CI: 1.05–1.62; *P* < .001). This was followed by a sharp increase to 3.90 in 2021 (APC: 12.73; 95% CI: 3.67–22.59; *P* = .008). It then decreased nonsignificantly to 3.35 in 2023 (APC: −6.61; 95% CI: −13.63 to 0.97; *P* = .08).

In the 55 to 64 years cohort, a gradual rise occurred from 1999 to 2018, with the rate increasing from 6.05 to 8.00 (APC: 1.51; 95% CI: 1.22–1.79; *P* < .001). This was followed by a sharp increase to 10.91 in 2021 (APC: 11.71; 95% CI: 3.91–20.09; *P* = .005). It then decreased nonsignificantly to 9.78 in 2023 (APC: −4.56; 95% CI: −10.80 to 2.12; *P* = .16).

In the 65 to 74 years cohort, the CMR remained stable from 1999 to 2018, fluctuating between 23.83 and 23.48 (APC: −0.04; 95% CI: −0.32 to 0.25; *P* = .79). This was followed by a sharp increase to 31.02 in 2021 (APC: 10.93; 95% CI: 2.77–19.74; *P* = .01). It then decreased nonsignificantly to 29.22 in 2023 (APC: −1.80; 95% CI: −8.17 to 5.01; *P* = .58).

In the 75 to 84 years cohort, the CMR declined steadily from 1999 to 2019, falling from 102.15 to 79.73 (APC: −0.89; 95% CI: −1.12 to −0.66; *P* < .001). This was followed by a significant increase until 2023 (APC: 4.70; 95% CI: 2.29–7.16; *P* < .001).

In the 85+ years cohort, the CMR declined steadily from 1999 to 2019, falling from 417.45 to 300.53 (APC: −1.33; 95% CI: −1.55 to −1.11; *P* < .001). This was followed by a significant increase to 353.06 in 2023 (APC: 3.52; 95% CI: 1.09–5.99; *P* = .006; Table S9).

**Figure 5.**
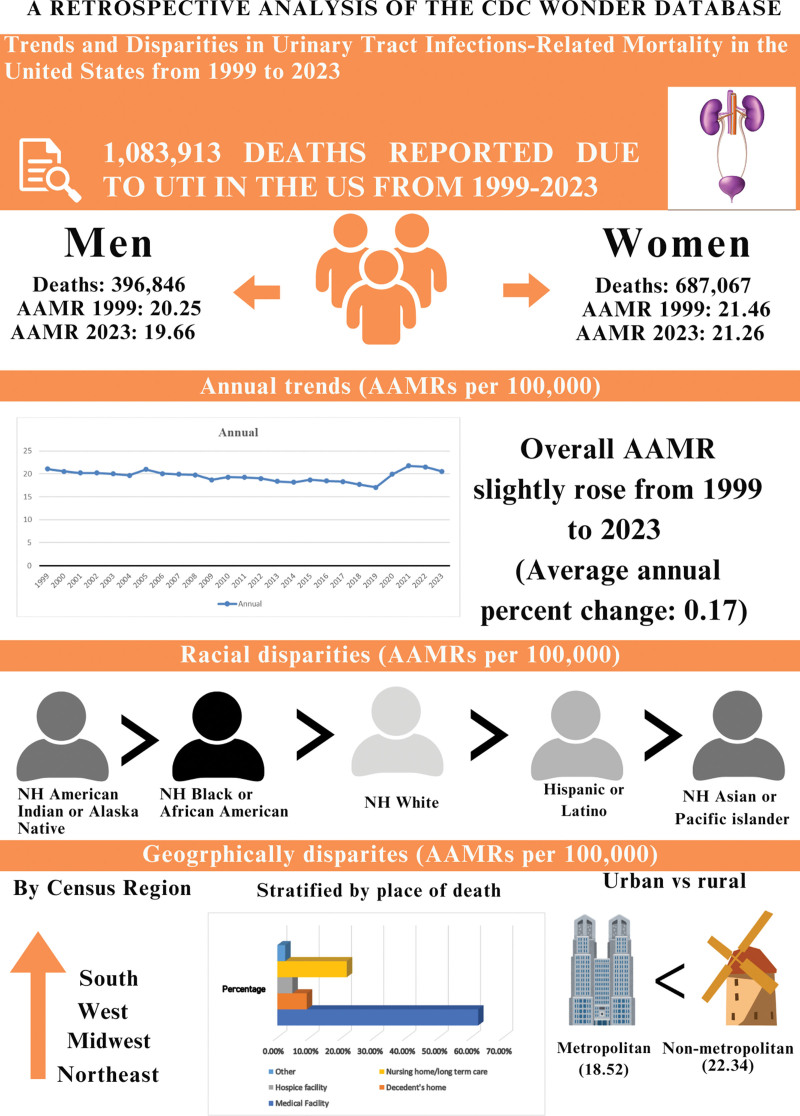
Central illustration, showing the summary of our results. AAMR = age-adjusted mortality rates, UTI = urinary tract infections.

Figure 5 depicts the summary of our results.

## 4. Discussion

From 1999 to 2023, UTI-related mortality in the United States underwent substantial change, with disparities by age, sex, race/ethnicity, and geography widening, a pattern that intensified during the COVID-19 period. AAMRs declined steadily from 1999 through 2018, rose sharply between 2019 and 2021, and then partially declined by 2023. Most UTI-related deaths occurred in medical facilities (62.25%), followed by nursing homes and long-term care facilities (LTCFs; 21.53%), reflecting that fatal UTI trajectories are usually managed in institutional rather than community settings. This finding underscores the central role of hospital and long-term care infrastructure, infection-prevention capacity, device stewardship, and timely sepsis care in determining outcomes during periods of system stress, a time when national surveillance documented pandemic-era surges in healthcare-associated infections, including catheter-associated urinary tract infection (CAUTI).^[16]^ Under-resourced or rural regions likely suffered disproportionate excess mortality during these surges because of limited intensive care unit/step-down capacity, staff shortages, and longer transfer times for time-sensitive conditions.^[16,17]^ The patterns we describe indicate an urgent need for scalable surge protocols, equitable resource allocation, and sustained investment in institutional infection-prevention and critical-care capacity to stabilize UTI outcomes across settings.^[16-19]^

UTI-related mortality rates declined from 1999 to 2018, likely reflecting advances in antimicrobial therapy, adoption of CAUTI-prevention bundles, improved chronic disease management, and stronger infection-prevention infrastructure in hospitals and LTCFs.^[20-23]^ Nevertheless, UTIs remained a leading cause of bacteremia and sepsis in older adults,^[19,24,25]^ and the syndrome’s clinical complexity, ranging from uncomplicated cystitis to multidrug-resistant, device-associated infections, requires early diagnosis and coordinated care across settings. The sharp rebound in mortality from 2019 to 2021 coincided with the COVID-19 pandemic, a period characterized by staffing shortages, increased device utilization, delayed presentations, and documented national increases in healthcare-associated infections (HAIs), including CAUTI.^[16]^ Hospitals and LTCFs operated under severe strain during this period, which likely increased risk for frail and catheterized patients. Although improvements began by 2023, mortality rates have not returned to pre-2018 baselines, highlighting the imperative for robust infection-prevention infrastructure, strengthened pandemic preparedness, and public education on UTI risk factors such as dehydration, inadequate perineal hygiene, and avoidable catheterization.^[20-25]^

Our analysis demonstrated that females persistently exhibited higher UTI-related mortality than males, a finding concordant with sex-linked differences in urinary-tract susceptibility and mucosal immunobiology. Key mechanisms include a shorter urethra, postmenopausal estrogen decline that weakens epithelial defenses, and sex-specific immune responses that increase recurrence and severity.^[25,26]^ In institutional settings, greater catheter exposure among older women and diagnostic ambiguity (asymptomatic bacteriuria vs infection) can delay therapy or prompt unnecessary antibiotic use, both plausibly linked to sepsis and worse outcomes.^[20-25]^ In device-heavy or obstructive settings, some male patients demonstrate comparable or greater severity, with higher catheter use and more frequent septic shock at presentation among very old men, indicating setting-specific risk modifiers.^[27]^ After a steady decline through 2018, mortality rose sharply in both sexes during 2019 to 2021, coincident with pandemic-era strain and surges in healthcare-associated infections, including CAUTI.^[16]^ Although rates eased thereafter, they remain above pre-2018 levels, supporting sex-aware interventions: catheter avoidance and early removal, competency-based insertion and maintenance, LTCF-focused infection prevention and stewardship, postmenopausal measures, and diagnostic stewardship to reduce missed infections and unnecessary antibiotics.^[20-25]^

Age-specific mortality rates show that older adults bore the highest burden throughout the study period, with peak rates in those ≥85 years. After 2018, mortality increased across all adult age groups. The fastest relative gains occurred in ages 25 to 34 (APC 8.39% to 2023), and ages 35 to 54 experienced a sharp 2020 to 2021 spike followed by partial easing, indicating a system-wide shift rather than an increase restricted to the elderly. Likely drivers include pandemic-era care delays, throughput constraints, and greater device exposure across age groups^[16,19]^ while frailty, multimorbidity, and long-term care residence maintained the highest absolute risk among the oldest adults.^[24]^

Significant racial disparities emerged. NH American Indian/Alaska Native populations had the highest UTI-related mortality. NH Asian/Pacific Islander groups had the lowest early rates but showed a renewed increase after 2018; NH Black populations also experienced elevated mortality across multiple years. These gaps align with evidence that severe sepsis incidence and outcomes vary by race/ethnicity because of differences in access and affordability constraints and higher baseline comorbidity burdens in some groups.^[28,29]^ During the COVID-19 period, facility-level strain and nationally documented surges in healthcare-associated infections (including CAUTI) likely exacerbated these disparities.^[16]^ Although rates eased after 2021, racial gaps persist. Mitigation requires timely access to evaluation and antimicrobials and targeted CAUTI-bundle adherence in high-burden facilities, consistent with LTCF and CAUTI-prevention literature.^[20-23]^

Geographic patterns were distinct. State-level burden clustered in the South and Appalachia, with the highest rates in Tennessee; Oklahoma, Mississippi, West Virginia, South Carolina, and Kentucky were also elevated. Hawaii had the lowest burden, and comparatively lower burdens were seen in Florida, Arizona, Nevada, Minnesota, and New Jersey. By urbanization, nonmetropolitan areas consistently exceeded metropolitan areas across the period, indicating a persistent rural–urban gap. Across census regions, the South maintained the highest overall levels, whereas the Northeast and Midwest showed a pre-2019 decline followed by a post-2018 increase; the West exhibited a similar decline-then-increase pattern through 2023. These geographic patterns align with broader evidence that rural systems face capacity constraints, longer transfer times, and limited specialist coverage for time-sensitive infections, and that pandemic-era infection-prevention strain and HAI surges (including CAUTI) were widespread, factors that can amplify baseline geographic differences.^[16,17]^

Collectively, these findings underscore the urgent need to reinforce infection-prevention capacity and promote equity in UTI management across institutional settings. Expanding tele-consultation and rapid escalation pathways for under-resourced rural hospitals and LTCFs could enable timely expertise on catheter decisions, early urosepsis recognition, and antimicrobial selection where specialist coverage is limited.^[17]^ In parallel, strengthening a national UTI/CAUTI surveillance backbone that links patient demographics, device use, resistance patterns, and outcomes would support near-real-time detection of disparities and targeted quality improvement, building on NHSN (National Healthcare Safety Network) HAI reporting.^[16]^ Complementing US work with international UTI burden and trend evidence can benchmark progress and surface generalizable levers, given global signals in incidence, severity, and resistance.^[5,25]^ These initiatives, together with sustained CAUTI-prevention bundles and stewardship in hospitals and LTCFs,^[20-23]^ will be essential to stabilize the 2019 to 2021 reversals, narrow geographic and racial gaps, and advance equitable infection outcomes across US regions.

## 5. Limitations

This study has several important limitations. First, using death-certificate data from CDC WONDER may have led to misclassification and to under- or over-attribution of UTI as the underlying versus a contributing cause of death; this misclassification could vary by region, place of death (hospital vs LTCFs), facility type, and patient demographics. Changes in hospice/LTCF coding during 2019 to 2021 may also have affected the reported distribution of place of death. Second, individual-level data were unavailable, so many potential confounders could not be captured for risk adjustment or causal inference, for example, urine culture and resistance profiles, catheter exposure and device-days, timing and appropriateness of antimicrobials, sepsis severity, long-term care residence, socioeconomic factors, and comorbidities or exposures (e.g., diabetes, chronic kidney disease, neurogenic bladder). Third, as an ecological, population-level analysis, our findings represent associations rather than causation and are not intended for individual-level prognosis or clinical decision-making. Fourth, temporal changes in diagnostic and coding practices, including the 2015 ICD-10 transition, evolving sepsis documentation, shifts between the terms “UTI” and “urosepsis,” and COVID-19–era testing and documentation, may have influenced cause-of-death attribution. Finally, regional healthcare infrastructure, transfer patterns, infection-prevention capacity, and staffing metrics were obtained from secondary sources rather than measured directly. Taken together, these limitations indicate that future research should use linked, patient-level datasets (clinical, microbiologic, device, and treatment-timing data) to validate our findings and to clarify the clinical determinants of UTI-related mortality.

## 6. Conclusion

This analysis offers a 25-year, comprehensive analysis of UTI-attributable mortality in the United States, documenting pronounced temporal trends and enduring disparities across sex, age, race/ethnicity, urbanization, and geographic region. A persistent rural–urban divide was evident: nonmetropolitan areas experienced consistently higher mortality than metropolitan areas, and this disparity widened during the 2019 to 2021 surge, suggesting structural vulnerability in decentralized hospital and long-term care systems. Following a downward trend until 2018, death rates surged during the COVID-19 period; the surge manifested as the highest absolute increases in older adults and the steepest relative increases among adults aged 25 to 34, with several racial and ethnic minority groups, rural communities, and Southern/Appalachian states disproportionately affected. Although rates had partially receded by 2023, substantial inequities persisted.

These results point to the need for policies that strengthen infection-prevention and sepsis capabilities across hospitals and LTCFs; bolster rural and state-level infrastructure (for example, tele-consultation, rapid escalation pathways, and coordinated transfer systems); and remedy structural barriers to timely diagnostics, antimicrobial access, and catheter stewardship. Sustained investment in public-health surveillance that links demographics, device exposure, resistance patterns, and outcomes, together with linked clinical datasets to complement mortality data, will be crucial not only for equitable preparedness and response during system stress, but also for real-time detection and targeted interventions to reduce preventable UTI mortality where gaps persist.

## Author contributions

**Conceptualization:** Shehdev Meghwar.

**Data curation:** Madina Memon.

**Formal analysis:** Riya Bhagwan.

**Supervision:** Shree Rath, Sabahat Ul Ain Munir Abbasi.

**Validation:** Shehdev Meghwar.

**Visualization:** Madina Memon.

**Writing – original draft:** Shehdev Meghwar, Sayed Maisum Mehdi Naqvi, Fnu Urooba, Chris Jerical, S. M. Aleem Hussain, Aseef Rehman, Vishan Das, Mohammed Hammad Jaber Amin.

**Writing – review & editing:** Aseef Rehman, Vishan Das, Mohammed Hammad Jaber Amin, Shree Rath, Sabahat Ul Ain Munir Abbasi.


















