# Supplementary material for: Trends and disparities in urinary tract infections-related mortality in the United States from 1999 to 2023: Insights from CDC WONDER
Source: Medicine (Baltimore). 2026 May 22;105(21):e49032. doi: 10.1097/MD.0000000000049032 (PMC13201035; doi:10.1097/MD.0000000000049032)
Supplement: Supplementary file 1 [file medi-105-e49032-s001.docx]

**Supplemental Table 1: Deaths per 1000,000 in the United States, 1999 to 2023**

| Year | Female | Male | NH American Indian or Alaska Native | NH White | NH Black or African American | Asian or Pacific Islander | Hispanic or Latino |
| --- | --- | --- | --- | --- | --- | --- | --- |
| 1999 | 24187 | 12741 | 116 | 30585 | 4433 | 394 | 1322 |
| 2000 | 23960 | 12498 | 145 | 30129 | 4309 | 432 | 1354 |
| 2001 | 24116 | 12382 | 158 | 30071 | 4310 | 483 | 1388 |
| 2002 | 24379 | 12588 | 150 | 30497 | 4308 | 490 | 1431 |
| 2003 | 24343 | 12928 | 165 | 30752 | 4238 | 473 | 1537 |
| 2004 | 24174 | 12988 | 156 | 30654 | 4133 | 563 | 1591 |
| 2005 | 26315 | 14134 | 193 | 33499 | 4343 | 610 | 1743 |
| 2006 | 25764 | 13849 | 165 | 32595 | 4252 | 649 | 1894 |
| 2007 | 26241 | 13884 | 197 | 33065 | 4267 | 687 | 1864 |
| 2008 | 26403 | 14308 | 223 | 33411 | 4255 | 776 | 1965 |
| 2009 | 25609 | 13831 | 213 | 32387 | 4056 | 779 | 1935 |
| 2010 | 26633 | 14615 | 230 | 33949 | 4067 | 756 | 2173 |
| 2011 | 27410 | 15079 | 259 | 34964 | 4214 | 810 | 2175 |
| 2012 | 27502 | 15369 | 269 | 35032 | 4258 | 839 | 2363 |
| 2013 | 27118 | 15302 | 252 | 34444 | 4266 | 903 | 2441 |
| 2014 | 27351 | 15529 | 284 | 34738 | 4297 | 903 | 2514 |
| 2015 | 28636 | 16509 | 298 | 36580 | 4476 | 924 | 2677 |
| 2016 | 28503 | 17004 | 299 | 36706 | 4591 | 974 | 2795 |
| 2017 | 28691 | 17447 | 310 | 37114 | 4580 | 1000 | 3010 |
| 2018 | 28087 | 17393 | 321 | 36612 | 4565 | 960 | 2926 |
| 2019 | 27288 | 17533 | 307 | 35974 | 4493 | 1034 | 2918 |
| 2020 | 32451 | 20855 | 438 | 41704 | 5806 | 1316 | 3921 |
| 2021 | 33915 | 21994 | 452 | 43732 | 5925 | 1304 | 4151 |
| 2022 | 34751 | 23552 | 450 | 45756 | 6133 | 1447 | 4158 |
| 2023 | 33240 | 22534 | 417 | 43580 | 5720 | 1462 | 4203 |
