# Supplementary material for: Trends and disparities in urinary tract infections-related mortality in the United States from 1999 to 2023: Insights from CDC WONDER
Source: Medicine (Baltimore). 2026 May 22;105(21):e49032. doi: 10.1097/MD.0000000000049032 (PMC13201035; doi:10.1097/MD.0000000000049032)
Supplement: Supplementary file 2 [file medi-105-e49032-s002.docx]

**Supplemental Table 2: Age-Adjusted Mortality Rates per 1000,000 in the United States, 1999 to 2023 by place of death.**

| Place of death | Deaths | Percentage |
| --- | --- | --- |
| Medical Facility | 674210 | 62.25% |
| 1. Medical Facility - Inpatient | 640444 | 95.00% |
| 1. Medical Facility - Outpatient or ER | 31537 | 4.67% |
| 1. Medical Facility - Dead on Arrival | 1354 | 0.20% |
| 1. Medical Facility - Status unknown | 875 | 0.13% |
| Decedent's home | 98876 | 9.10% |
| Hospice facility | 50625 | 4.68% |
| Nursing home | 233139 | 21.53% |
| Other | 26410 | 2.44% |
| Place of death unknown | 2417 | 0.22% |
