# Supplementary material for: Trends and disparities in urinary tract infections-related mortality in the United States from 1999 to 2023: Insights from CDC WONDER
Source: Medicine (Baltimore). 2026 May 22;105(21):e49032. doi: 10.1097/MD.0000000000049032 (PMC13201035; doi:10.1097/MD.0000000000049032)
Supplement: Supplementary file 3 [file medi-105-e49032-s003.docx]

**Supplemental Table 3: Overall Age-Adjusted Mortality Rates per 1000,000 in the United States, 1999 to 2023**

| Age Adjusted Rate (95% CI) OVERALL | | | |
| --- | --- | --- | --- |
| Year | Age Adjusted Rate | Age Adjusted Rate Lower 95% Confidence Interval | Age Adjusted Rate Upper 95% Confidence Interval |
| 1999 | 21.0415 | 20.8268 | 21.2561 |
| 2000 | 20.4934 | 20.283 | 20.7037 |
| 2001 | 20.174 | 19.967 | 20.381 |
| 2002 | 20.1686 | 19.963 | 20.3743 |
| 2003 | 19.976 | 19.7731 | 20.1789 |
| 2004 | 19.6325 | 19.4328 | 19.8322 |
| 2005 | 20.9304 | 20.7262 | 21.1346 |
| 2006 | 20.0438 | 19.8461 | 20.2415 |
| 2007 | 19.8729 | 19.678 | 20.0679 |
| 2008 | 19.7432 | 19.5508 | 19.9356 |
| 2009 | 18.7046 | 18.5192 | 18.89 |
| 2010 | 19.2474 | 19.0607 | 19.4342 |
| 2011 | 19.2124 | 19.0284 | 19.3964 |
| 2012 | 18.9497 | 18.7688 | 19.1306 |
| 2013 | 18.3123 | 18.1363 | 18.4882 |
| 2014 | 18.1514 | 17.9778 | 18.3249 |
| 2015 | 18.6671 | 18.4931 | 18.8412 |
| 2016 | 18.4464 | 18.2751 | 18.6178 |
| 2017 | 18.2873 | 18.1186 | 18.456 |
| 2018 | 17.6315 | 17.4679 | 17.7951 |
| 2019 | 17.0212 | 16.8621 | 17.1802 |
| 2020 | 19.8986 | 19.7281 | 20.0691 |
| 2021 | 21.7295 | 21.5475 | 21.9114 |
| 2022 | 21.4446 | 21.2691 | 21.6202 |
| 2023 | 20.5005 | 20.329 | 20.6721 |
