# Supplementary material for: Trends and disparities in urinary tract infections-related mortality in the United States from 1999 to 2023: Insights from CDC WONDER
Source: Medicine (Baltimore). 2026 May 22;105(21):e49032. doi: 10.1097/MD.0000000000049032 (PMC13201035; doi:10.1097/MD.0000000000049032)
Supplement: Supplementary file 4 [file medi-105-e49032-s004.docx]

**Supplemental Table 4: Sex‐Stratified Age-Adjusted Mortality Rates per 1000,000 in the United States, 1999 to 2023**

| Age Adjusted Rate (95% CI) SEX (Females) | | | |
| --- | --- | --- | --- |
| Year | Age Adjusted Rate | Age Adjusted Rate Lower 95% Confidence Interval | Age Adjusted Rate Upper 95% Confidence Interval |
| 1999 | 21.4557 | 21.1839 | 21.7275 |
| 2000 | 21.0098 | 20.7423 | 21.2774 |
| 2001 | 20.9075 | 20.6421 | 21.1729 |
| 2002 | 20.9251 | 20.661 | 21.1893 |
| 2003 | 20.6255 | 20.3649 | 20.8861 |
| 2004 | 20.3225 | 20.0647 | 20.5804 |
| 2005 | 21.7209 | 21.4566 | 21.9852 |
| 2006 | 20.9369 | 20.679 | 21.1948 |
| 2007 | 20.9828 | 20.7263 | 21.2393 |
| 2008 | 20.7277 | 20.4749 | 20.9806 |
| 2009 | 19.7739 | 19.5285 | 20.0194 |
| 2010 | 20.2717 | 20.0246 | 20.5188 |
| 2011 | 20.317 | 20.0723 | 20.5617 |
| 2012 | 20.0832 | 19.8413 | 20.3251 |
| 2013 | 19.4772 | 19.2408 | 19.7135 |
| 2014 | 19.3635 | 19.1295 | 19.5976 |
| 2015 | 19.9059 | 19.6708 | 20.141 |
| 2016 | 19.5084 | 19.2775 | 19.7394 |
| 2017 | 19.2807 | 19.0534 | 19.5079 |
| 2018 | 18.5363 | 18.3157 | 18.7569 |
| 2019 | 17.752 | 17.5379 | 17.966 |
| 2020 | 20.8733 | 20.6425 | 21.1041 |
| 2021 | 22.9436 | 22.6963 | 23.1909 |
| 2022 | 22.0983 | 21.8626 | 22.334 |
| 2023 | 21.3162 | 21.0847 | 21.5478 |

| Age Adjusted Rate (95% CI) SEX (Males) | | | |
| --- | --- | --- | --- |
| Year | Age Adjusted Rate | Age Adjusted Rate Lower 95% Confidence Interval | Age Adjusted Rate Upper 95% Confidence Interval |
| 1999 | 20.2467 | 19.8876 | 20.6059 |
| 2000 | 19.5806 | 19.2304 | 19.9307 |
| 2001 | 18.8026 | 18.4649 | 19.1403 |
| 2002 | 18.8176 | 18.4825 | 19.1527 |
| 2003 | 18.7117 | 18.3834 | 19.0401 |
| 2004 | 18.5137 | 18.1899 | 18.8374 |
| 2005 | 19.5677 | 19.2403 | 19.8952 |
| 2006 | 18.6861 | 18.3709 | 19.0013 |
| 2007 | 18.1928 | 17.8867 | 18.4989 |
| 2008 | 18.2225 | 17.9207 | 18.5244 |
| 2009 | 17.1353 | 16.8467 | 17.4239 |
| 2010 | 17.7409 | 17.4504 | 18.0315 |
| 2011 | 17.6341 | 17.3499 | 17.9183 |
| 2012 | 17.4026 | 17.1246 | 17.6806 |
| 2013 | 16.7315 | 16.4634 | 16.9995 |
| 2014 | 16.5017 | 16.2392 | 16.7642 |
| 2015 | 16.9969 | 16.7345 | 17.2593 |
| 2016 | 17.0952 | 16.8351 | 17.3553 |
| 2017 | 17.0221 | 16.7664 | 17.2779 |
| 2018 | 16.4712 | 16.2235 | 16.7189 |
| 2019 | 16.1499 | 15.908 | 16.3919 |
| 2020 | 18.72 | 18.4628 | 18.9772 |
| 2021 | 20.3371 | 20.0628 | 20.6114 |
| 2022 | 20.928 | 20.6562 | 21.1998 |
| 2023 | 19.6637 | 19.4022 | 19.9251 |
