# Supplementary material for: Trends and disparities in urinary tract infections-related mortality in the United States from 1999 to 2023: Insights from CDC WONDER
Source: Medicine (Baltimore). 2026 May 22;105(21):e49032. doi: 10.1097/MD.0000000000049032 (PMC13201035; doi:10.1097/MD.0000000000049032)
Supplement: Supplementary file 5 [file medi-105-e49032-s005.docx]

**Supplemental Table 5: Race‐Stratified Age-Adjusted Mortality Rates per 1000,000 in the United States, 1999 to 2023**

| Age Adjusted Rate (95% CI) AMERICAN INDIAN OR ALASKA NATIVE | | | |
| --- | --- | --- | --- |
| Year | Age Adjusted Rate | Age Adjusted Rate Lower 95% Confidence Interval | Age Adjusted Rate Upper 95% Confidence Interval |
| 1999 | 18.541 | 14.9769 | 22.1051 |
| 2000 | 21.7919 | 18.0914 | 25.4924 |
| 2001 | 22.6002 | 18.9163 | 26.2841 |
| 2002 | 20.7508 | 17.2525 | 24.249 |
| 2003 | 22.6886 | 19.0515 | 26.3258 |
| 2004 | 19.4323 | 16.194 | 22.6705 |
| 2005 | 24.7074 | 21.0275 | 28.3873 |
| 2006 | 20.5006 | 17.2007 | 23.8005 |
| 2007 | 23.7456 | 20.239 | 27.2522 |
| 2008 | 25.2378 | 21.7315 | 28.744 |
| 2009 | 24.0225 | 20.6202 | 27.4248 |
| 2010 | 24.2992 | 20.9769 | 27.6216 |
| 2011 | 26.6274 | 23.2309 | 30.0238 |
| 2012 | 25.0263 | 21.8893 | 28.1632 |
| 2013 | 22.2661 | 19.3905 | 25.1418 |
| 2014 | 23.3415 | 20.5107 | 26.1722 |
| 2015 | 23.2208 | 20.4747 | 25.9669 |
| 2016 | 22.0217 | 19.4326 | 24.6108 |
| 2017 | 22.2955 | 19.7309 | 24.8602 |
| 2018 | 21.417 | 19.0024 | 23.8317 |
| 2019 | 20.1049 | 17.7967 | 22.4132 |
| 2020 | 26.4796 | 23.9304 | 29.0288 |
| 2021 | 30.5011 | 27.6125 | 33.3898 |
| 2022 | 27.9681 | 25.3302 | 30.6061 |
| 2023 | 25.1723 | 22.7116 | 27.6329 |

| Age Adjusted Rate (95% CI) ASIAN OR PACIFIC ISLANDER | | | |
| --- | --- | --- | --- |
| Year | Age Adjusted Rate | Age Adjusted Rate Lower 95% Confidence Interval | Age Adjusted Rate Upper 95% Confidence Interval |
| 1999 | 11.7898 | 10.58 | 12.9995 |
| 2000 | 11.868 | 10.7114 | 13.0245 |
| 2001 | 12.1171 | 11.0006 | 13.2335 |
| 2002 | 11.7217 | 10.6515 | 12.7919 |
| 2003 | 10.484 | 9.5115 | 11.4566 |
| 2004 | 11.9636 | 10.9502 | 12.9771 |
| 2005 | 12.1217 | 11.1384 | 13.1051 |
| 2006 | 11.8875 | 10.9522 | 12.8228 |
| 2007 | 11.8984 | 10.9888 | 12.808 |
| 2008 | 12.7552 | 11.8422 | 13.6682 |
| 2009 | 12.0175 | 11.1593 | 12.8758 |
| 2010 | 11.2503 | 10.4356 | 12.065 |
| 2011 | 10.9412 | 10.177 | 11.7054 |
| 2012 | 10.4207 | 9.7055 | 11.136 |
| 2013 | 10.4015 | 9.7144 | 11.0886 |
| 2014 | 9.6401 | 9.0034 | 10.2768 |
| 2015 | 9.283 | 8.6785 | 9.8875 |
| 2016 | 9.1698 | 8.5875 | 9.7521 |
| 2017 | 8.7749 | 8.2258 | 9.3241 |
| 2018 | 7.9363 | 7.4293 | 8.4432 |
| 2019 | 8.1557 | 7.654 | 8.6573 |
| 2020 | 9.8038 | 9.2696 | 10.3379 |
| 2021 | 10.0195 | 9.4707 | 10.5683 |
| 2022 | 10.314 | 9.779 | 10.849 |
| 2023 | 10.0616 | 9.5431 | 10.5802 |

| Age Adjusted Rate (95% CI) BLACK OR AFRICAN AMERICAN | | | |
| --- | --- | --- | --- |
| Year | Age Adjusted Rate | Age Adjusted Rate Lower 95% Confidence Interval | Age Adjusted Rate Upper 95% Confidence Interval |
| 1999 | 31.9163 | 30.9691 | 32.8636 |
| 2000 | 30.6832 | 29.7599 | 31.6065 |
| 2001 | 30.2678 | 29.3554 | 31.1801 |
| 2002 | 29.9942 | 29.0883 | 30.9 |
| 2003 | 29.0149 | 28.1302 | 29.8997 |
| 2004 | 27.8803 | 27.0184 | 28.7422 |
| 2005 | 28.4667 | 27.6078 | 29.3255 |
| 2006 | 27.2218 | 26.3907 | 28.0529 |
| 2007 | 26.5365 | 25.7276 | 27.3454 |
| 2008 | 25.8117 | 25.0233 | 26.6001 |
| 2009 | 23.8221 | 23.0763 | 24.568 |
| 2010 | 23.147 | 22.4222 | 23.8718 |
| 2011 | 23.1959 | 22.4822 | 23.9095 |
| 2012 | 22.5396 | 21.8494 | 23.2297 |
| 2013 | 21.6659 | 21.0022 | 22.3297 |
| 2014 | 21.1112 | 20.4668 | 21.7556 |
| 2015 | 21.0833 | 20.452 | 21.7146 |
| 2016 | 20.9959 | 20.3751 | 21.6166 |
| 2017 | 20.1326 | 19.5362 | 20.7291 |
| 2018 | 19.5656 | 18.9858 | 20.1453 |
| 2019 | 18.4935 | 17.9409 | 19.0461 |
| 2020 | 23.1939 | 22.5844 | 23.8034 |
| 2021 | 24.3173 | 23.6799 | 24.9547 |
| 2022 | 24.349 | 23.7248 | 24.9731 |
| 2023 | 22.2731 | 21.6808 | 22.8654 |

| Age Adjusted Rate (95% CI) WHITE | | | |
| --- | --- | --- | --- |
| Year | Age Adjusted Rate | Age Adjusted Rate Lower 95% Confidence Interval | Age Adjusted Rate Upper 95% Confidence Interval |
| 1999 | 20.3434 | 20.1153 | 20.5716 |
| 2000 | 19.8034 | 19.5796 | 20.0271 |
| 2001 | 19.5122 | 19.2915 | 19.7329 |
| 2002 | 19.575 | 19.3551 | 19.7949 |
| 2003 | 19.4463 | 19.2288 | 19.6639 |
| 2004 | 19.1899 | 18.9747 | 19.405 |
| 2005 | 20.6485 | 20.4269 | 20.8701 |
| 2006 | 19.7238 | 19.509 | 19.9385 |
| 2007 | 19.66 | 19.4471 | 19.8728 |
| 2008 | 19.5258 | 19.3154 | 19.7362 |
| 2009 | 18.6226 | 18.4183 | 18.8268 |
| 2010 | 19.327 | 19.1195 | 19.5345 |
| 2011 | 19.3862 | 19.1808 | 19.5916 |
| 2012 | 19.1427 | 18.9397 | 19.3456 |
| 2013 | 18.5029 | 18.3049 | 18.7009 |
| 2014 | 18.4478 | 18.2511 | 18.6446 |
| 2015 | 19.1256 | 18.9268 | 19.3244 |
| 2016 | 18.9779 | 18.7805 | 19.1752 |
| 2017 | 18.8886 | 18.6937 | 19.0836 |
| 2018 | 18.3273 | 18.137 | 18.5176 |
| 2019 | 17.774 | 17.5876 | 17.9604 |
| 2020 | 20.4291 | 20.2301 | 20.6281 |
| 2021 | 22.7351 | 22.5191 | 22.9511 |
| 2022 | 22.4665 | 22.2578 | 22.6752 |
| 2023 | 21.5941 | 21.3886 | 21.7996 |

| Age Adjusted Rate (95% CI) HISPANICS | | | |
| --- | --- | --- | --- |
| Year | Age Adjusted Rate | Age Adjusted Rate Lower 95% Confidence Interval | Age Adjusted Rate Upper 95% Confidence Interval |
| 1999 | 17.5501 | 16.5672 | 18.5329 |
| 2000 | 17.1669 | 16.2198 | 18.114 |
| 2001 | 16.5704 | 15.6687 | 17.4721 |
| 2002 | 16.1893 | 15.3176 | 17.061 |
| 2003 | 16.2023 | 15.3605 | 17.0441 |
| 2004 | 16.1825 | 15.3561 | 17.009 |
| 2005 | 16.7476 | 15.933 | 17.5623 |
| 2006 | 17.0953 | 16.298 | 17.8926 |
| 2007 | 16.0207 | 15.2691 | 16.7723 |
| 2008 | 15.8953 | 15.1684 | 16.6222 |
| 2009 | 14.6616 | 13.9863 | 15.3368 |
| 2010 | 15.9051 | 15.2145 | 16.5956 |
| 2011 | 14.5892 | 13.9584 | 15.2199 |
| 2012 | 14.9879 | 14.367 | 15.6088 |
| 2013 | 14.6572 | 14.0616 | 15.2529 |
| 2014 | 14.0034 | 13.4418 | 14.5651 |
| 2015 | 14.0654 | 13.5195 | 14.6114 |
| 2016 | 13.9735 | 13.4428 | 14.5041 |
| 2017 | 14.0888 | 13.5725 | 14.605 |
| 2018 | 13.1599 | 12.6713 | 13.6484 |
| 2019 | 12.5626 | 12.0959 | 13.0292 |
| 2020 | 15.9603 | 15.4488 | 16.4718 |
| 2021 | 16.763 | 16.2372 | 17.2887 |
| 2022 | 16.0753 | 15.5742 | 16.5765 |
| 2023 | 15.821 | 15.3295 | 16.3124 |
