# Supplementary material for: Trends and disparities in urinary tract infections-related mortality in the United States from 1999 to 2023: Insights from CDC WONDER
Source: Medicine (Baltimore). 2026 May 22;105(21):e49032. doi: 10.1097/MD.0000000000049032 (PMC13201035; doi:10.1097/MD.0000000000049032)
Supplement: Supplementary file 6 [file medi-105-e49032-s006.docx]

**Supplemental Table 6: Age-Adjusted Mortality Rates per 1000,000 in the United States, 1999 to 2020 by State.**

|  | 1999-2020 | | | 2021-2023 | | |
| --- | --- | --- | --- | --- | --- | --- |
| State | Age Adjusted Rate | Age Adjusted Rate Lower 95% Confidence Interval | Age Adjusted Rate Upper 95% Confidence Interval | Age Adjusted Rate | Age Adjusted Rate Lower 95% Confidence Interval | Age Adjusted Rate Upper 95% Confidence Interval |
| Alabama | 21.7619 | 21.4209 | 22.1029 | 20.58 | 19.77 | 21.42 |
| Alaska | 18.5572 | 17.365 | 19.7493 | 20.47 | 17.81 | 23.43 |
| Arizona | 12.9565 | 12.7305 | 13.1824 | 14.87 | 14.32 | 15.44 |
| Arkansas | 22.1504 | 21.723 | 22.5778 | 23.9 | 22.78 | 25.07 |
| California | 20.8695 | 20.7441 | 20.9948 | 20.24 | 19.94 | 20.55 |
| Colorado | 17.5934 | 17.2652 | 17.9217 | 25.28 | 24.37 | 26.22 |
| Connecticut | 17.4481 | 17.1243 | 17.7718 | 16.35 | 15.55 | 17.19 |
| Delaware | 18.9704 | 18.2542 | 19.6866 | 19 | 17.36 | 20.78 |
| District of Columbia | 22.1424 | 21.1406 | 23.1443 | 19.93 | 17.56 | 22.55 |
| Florida | 12.7193 | 12.6057 | 12.833 | 18.89 | 18.56 | 19.23 |
| Georgia | 18.6974 | 18.4435 | 18.9514 | 19.31 | 18.72 | 19.92 |
| Hawaii | 11.1528 | 10.7263 | 11.5793 | 9.11 | 8.16 | 10.17 |
| Idaho | 19.5353 | 18.9495 | 20.121 | 24.26 | 22.77 | 25.84 |
| Illinois | 17.6417 | 17.4552 | 17.8283 | 17.41 | 16.94 | 17.89 |
| Indiana | 21.798 | 21.5057 | 22.0902 | 25.29 | 24.5 | 26.1 |
| Iowa | 18.7297 | 18.3746 | 19.0848 | 22.97 | 21.94 | 24.04 |
| Kansas | 16.3388 | 15.9732 | 16.7045 | 20.74 | 19.68 | 21.85 |
| Kentucky | 26.7733 | 26.3721 | 27.1744 | 34.56 | 33.43 | 35.73 |
| Louisiana | 20.8268 | 20.4706 | 21.1829 | 21.81 | 20.91 | 22.75 |
| Maine | 18.2315 | 17.6978 | 18.7651 | 16.61 | 15.37 | 17.96 |
| Maryland | 20.8554 | 20.5444 | 21.1664 | 24.16 | 23.35 | 24.98 |
| Massachusetts | 17.2432 | 17.0017 | 17.4848 | 18.84 | 18.2 | 19.5 |
| Michigan | 17.7799 | 17.573 | 17.9869 | 18.64 | 18.11 | 19.19 |
| Minnesota | 15.2704 | 15.0076 | 15.5332 | 20.84 | 20.08 | 21.62 |
| Mississippi | 27.9741 | 27.4698 | 28.4783 | 39.45 | 37.95 | 41 |
| Missouri | 17.566 | 17.3037 | 17.8283 | 18.64 | 17.95 | 19.34 |
| Montana | 17.5787 | 16.9472 | 18.2101 | 22.05 | 20.34 | 23.9 |
| Nebraska | 17.1281 | 16.6668 | 17.5895 | 23.34 | 21.96 | 24.79 |
| Nevada | 14.4119 | 13.9973 | 14.8264 | 19.3 | 18.26 | 20.38 |
| New Hampshire | 17.3324 | 16.7691 | 17.8956 | 19.53 | 18.11 | 21.05 |
| New Jersey | 15.0869 | 14.8866 | 15.2872 | 16.15 | 15.63 | 16.68 |
| New Mexico | 18.1723 | 17.6869 | 18.6577 | 18.96 | 17.78 | 20.2 |
| New York | 16.0758 | 15.9367 | 16.215 | 16.61 | 16.25 | 16.97 |
| North Carolina | 25.4231 | 25.1548 | 25.6914 | 22.89 | 22.29 | 23.5 |
| North Dakota | 15.8554 | 15.1575 | 16.5533 | 19 | 17.05 | 21.15 |
| Ohio | 20.0153 | 19.8149 | 20.2156 | 18.93 | 18.43 | 19.45 |
| Oklahoma | 28.9143 | 28.473 | 29.3557 | 49.99 | 48.51 | 51.51 |
| Oregon | 18.9144 | 18.5755 | 19.2533 | 26.41 | 25.43 | 27.43 |
| Pennsylvania | 16.2907 | 16.1295 | 16.4519 | 18.9 | 18.45 | 19.37 |
| Rhode Island | 25.8344 | 25.1218 | 26.547 | 18.6 | 17.05 | 20.27 |
| South Carolina | 26.977 | 26.584 | 27.37 | 27.44 | 26.53 | 28.38 |
| South Dakota | 19.0329 | 18.3277 | 19.738 | 30.25 | 27.95 | 32.72 |
| Tennessee | 30.4767 | 30.1218 | 30.8316 | 28.61 | 27.78 | 29.47 |
| Texas | 22.9595 | 22.7857 | 23.1333 | 24.84 | 24.41 | 25.26 |
| Utah | 21.6982 | 21.1534 | 22.243 | 25.13 | 23.78 | 26.55 |
| Vermont | 20.6696 | 19.805 | 21.5341 | 23.43 | 21.21 | 25.88 |
| Virginia | 19.9661 | 19.7016 | 20.2305 | 19.66 | 19.04 | 20.29 |
| Washington | 23.6363 | 23.3308 | 23.9418 | 28.23 | 27.43 | 29.05 |
| West Virginia | 28.4852 | 27.9073 | 29.063 | 30.68 | 29.13 | 32.32 |
| Wisconsin | 17.0279 | 16.7657 | 17.2901 | 21.18 | 20.44 | 21.95 |
| Wyoming | 20.2325 | 19.2372 | 21.2279 | 30.62 | 27.7 | 33.8 |
