# Supplementary material for: Trends and disparities in urinary tract infections-related mortality in the United States from 1999 to 2023: Insights from CDC WONDER
Source: Medicine (Baltimore). 2026 May 22;105(21):e49032. doi: 10.1097/MD.0000000000049032 (PMC13201035; doi:10.1097/MD.0000000000049032)
Supplement: Supplementary file 7 [file medi-105-e49032-s007.docx]

**Supplemental Table 7: Urbanization‐Stratified Age-Adjusted Mortality Rates per 1000,000 in the United States, 1999 to 2020**

| Age Adjusted Rate (95% CI) METROPOLITAN | | | |
| --- | --- | --- | --- |
| Year | Age Adjusted Rate | Age Adjusted Rate Lower 95% Confidence Interval | Age Adjusted Rate Upper 95% Confidence Interval |
| 1999 | 20.7396 | 20.5021 | 20.9772 |
| 2000 | 20.1305 | 19.8985 | 20.3626 |
| 2001 | 19.8032 | 19.5751 | 20.0313 |
| 2002 | 19.6254 | 19.4001 | 19.8507 |
| 2003 | 19.4292 | 19.2071 | 19.6513 |
| 2004 | 19.1952 | 18.9762 | 19.4143 |
| 2005 | 20.2739 | 20.0513 | 20.4964 |
| 2006 | 19.4618 | 19.2461 | 19.6775 |
| 2007 | 19.2118 | 19 | 19.4236 |
| 2008 | 19.0772 | 18.8682 | 19.2862 |
| 2009 | 18.0716 | 17.8704 | 18.2729 |
| 2010 | 18.7148 | 18.5115 | 18.9181 |
| 2011 | 18.5177 | 18.3187 | 18.7167 |
| 2012 | 18.2301 | 18.0347 | 18.4256 |
| 2013 | 17.6704 | 17.48 | 17.8607 |
| 2014 | 17.4444 | 17.2576 | 17.6312 |
| 2015 | 17.8064 | 17.6197 | 17.9931 |
| 2016 | 17.6504 | 17.4665 | 17.8343 |
| 2017 | 17.5545 | 17.3733 | 17.7356 |
| 2018 | 16.7607 | 16.586 | 16.9353 |
| 2019 | 16.1819 | 16.0123 | 16.3515 |
| 2020 | 18.9838 | 18.8014 | 19.1662 |

| Age Adjusted Rate (95% CI) NONMETROPOLITAN | | | |
| --- | --- | --- | --- |
| Year | Age Adjusted Rate | Age Adjusted Rate Lower 95% Confidence Interval | Age Adjusted Rate Upper 95% Confidence Interval |
| 1999 | 22.4243 | 21.9208 | 22.9279 |
| 2000 | 21.8969 | 21.4022 | 22.3917 |
| 2001 | 21.7859 | 21.2932 | 22.2787 |
| 2002 | 22.4147 | 21.9166 | 22.9128 |
| 2003 | 22.3402 | 21.8454 | 22.8351 |
| 2004 | 21.594 | 21.108 | 22.0799 |
| 2005 | 23.8252 | 23.318 | 24.3323 |
| 2006 | 22.703 | 22.2111 | 23.1949 |
| 2007 | 22.728 | 22.2392 | 23.2168 |
| 2008 | 22.7491 | 22.2628 | 23.2355 |
| 2009 | 21.728 | 21.2542 | 22.2017 |
| 2010 | 21.7982 | 21.3267 | 22.2696 |
| 2011 | 22.4712 | 21.9957 | 22.9466 |
| 2012 | 22.4007 | 21.9302 | 22.8712 |
| 2013 | 21.5364 | 21.0781 | 21.9947 |
| 2014 | 21.471 | 21.0138 | 21.9282 |
| 2015 | 22.9267 | 22.4579 | 23.3955 |
| 2016 | 22.4463 | 21.9848 | 22.9078 |
| 2017 | 21.9312 | 21.4793 | 22.3831 |
| 2018 | 21.9394 | 21.4908 | 22.388 |
| 2019 | 21.2664 | 20.8265 | 21.7062 |
| 2020 | 24.782 | 24.3099 | 25.2542 |
