# Supplementary material for: Trends and disparities in urinary tract infections-related mortality in the United States from 1999 to 2023: Insights from CDC WONDER
Source: Medicine (Baltimore). 2026 May 22;105(21):e49032. doi: 10.1097/MD.0000000000049032 (PMC13201035; doi:10.1097/MD.0000000000049032)
Supplement: Supplementary file 8 [file medi-105-e49032-s008.docx]

**Supplemental Table 8: Region‐Stratified Age-Adjusted Mortality Rates per 1000,000 in the United States, 1999 to 2023**

| Age Adjusted Rate (95% CI) NORTHEAST | | | |
| --- | --- | --- | --- |
| Year | Age Adjusted Rate | Age Adjusted Rate Lower 95% Confidence Interval | Age Adjusted Rate Upper 95% Confidence Interval |
| 1999 | 17.9121 | 17.4855 | 18.3386 |
| 2000 | 17.3878 | 16.9706 | 17.8049 |
| 2001 | 17.3395 | 16.9258 | 17.7532 |
| 2002 | 17.6221 | 17.2077 | 18.0365 |
| 2003 | 17.0683 | 16.6628 | 17.4738 |
| 2004 | 17.505 | 17.0962 | 17.9139 |
| 2005 | 18.3623 | 17.9468 | 18.7778 |
| 2006 | 17.4433 | 17.0407 | 17.846 |
| 2007 | 17.2511 | 16.8534 | 17.6488 |
| 2008 | 17.2498 | 16.8545 | 17.6451 |
| 2009 | 16.0334 | 15.6541 | 16.4126 |
| 2010 | 16.9447 | 16.5564 | 17.3331 |
| 2011 | 17.5392 | 17.1467 | 17.9316 |
| 2012 | 16.7957 | 16.4138 | 17.1777 |
| 2013 | 16.1166 | 15.7452 | 16.4881 |
| 2014 | 16.0033 | 15.6353 | 16.3712 |
| 2015 | 16.1034 | 15.7363 | 16.4705 |
| 2016 | 15.4353 | 15.0777 | 15.793 |
| 2017 | 14.9407 | 14.5932 | 15.2881 |
| 2018 | 14.668 | 14.3262 | 15.0098 |
| 2019 | 13.939 | 13.6093 | 14.2688 |
| 2020 | 16.3396 | 15.9835 | 16.6956 |
| 2021 | 17.3701 | 16.9984 | 17.7417 |
| 2022 | 17.9541 | 17.5847 | 18.3235 |
| 2023 | 17.153 | 16.7916 | 17.5143 |

| Age Adjusted Rate (95% CI) MIDWEST | | | |
| --- | --- | --- | --- |
| Year | Age Adjusted Rate | Age Adjusted Rate Lower 95% Confidence Interval | Age Adjusted Rate Upper 95% Confidence Interval |
| 1999 | 19.8778 | 19.4554 | 20.3002 |
| 2000 | 18.7327 | 18.3242 | 19.1412 |
| 2001 | 18.5322 | 18.1285 | 18.9359 |
| 2002 | 18.6486 | 18.2458 | 19.0513 |
| 2003 | 18.4648 | 18.0666 | 18.863 |
| 2004 | 18.2294 | 17.8356 | 18.6231 |
| 2005 | 20.0702 | 19.6599 | 20.4804 |
| 2006 | 19.075 | 18.6786 | 19.4714 |
| 2007 | 19.1919 | 18.7964 | 19.5874 |
| 2008 | 19.1319 | 18.74 | 19.5237 |
| 2009 | 17.6863 | 17.312 | 18.0606 |
| 2010 | 18.0859 | 17.7089 | 18.4629 |
| 2011 | 18.6683 | 18.2888 | 19.0478 |
| 2012 | 18.1012 | 17.7303 | 18.4721 |
| 2013 | 17.0527 | 16.6955 | 17.4099 |
| 2014 | 17.276 | 16.9182 | 17.6338 |
| 2015 | 17.6106 | 17.2514 | 17.9697 |
| 2016 | 17.356 | 17.0024 | 17.7096 |
| 2017 | 17.2491 | 16.8999 | 17.5984 |
| 2018 | 16.8244 | 16.4821 | 17.1667 |
| 2019 | 16.3081 | 15.9736 | 16.6425 |
| 2020 | 19.5642 | 19.1986 | 19.9298 |
| 2021 | 20.79 | 20.4041 | 21.176 |
| 2022 | 20.2756 | 19.9039 | 20.6472 |
| 2023 | 19.1031 | 18.7421 | 19.4642 |

| Age Adjusted Rate (95% CI) SOUTH | | | |
| --- | --- | --- | --- |
| Year | Age Adjusted Rate | Age Adjusted Rate Lower 95% Confidence Interval | Age Adjusted Rate Upper 95% Confidence Interval |
| 1999 | 23.9703 | 23.5802 | 24.3605 |
| 2000 | 23.6776 | 23.2928 | 24.0625 |
| 2001 | 23.1445 | 22.7666 | 23.5224 |
| 2002 | 22.9028 | 22.5292 | 23.2764 |
| 2003 | 22.4539 | 22.0869 | 22.8209 |
| 2004 | 21.6278 | 21.2704 | 21.9852 |
| 2005 | 23.0887 | 22.724 | 23.4535 |
| 2006 | 21.717 | 21.3675 | 22.0666 |
| 2007 | 21.5309 | 21.1871 | 21.8747 |
| 2008 | 21.1556 | 20.8187 | 21.4924 |
| 2009 | 20.3906 | 20.0634 | 20.7178 |
| 2010 | 20.6807 | 20.3545 | 21.0069 |
| 2011 | 20.0836 | 19.7676 | 20.3996 |
| 2012 | 20.4033 | 20.0894 | 20.7172 |
| 2013 | 19.8329 | 19.5273 | 20.1385 |
| 2014 | 19.5642 | 19.2647 | 19.8638 |
| 2015 | 20.4956 | 20.1937 | 20.7975 |
| 2016 | 20.513 | 20.2146 | 20.8113 |
| 2017 | 20.4756 | 20.1811 | 20.77 |
| 2018 | 19.5044 | 19.2216 | 19.7873 |
| 2019 | 19.24 | 18.9625 | 19.5175 |
| 2020 | 22.6041 | 22.307 | 22.9013 |
| 2021 | 24.646 | 24.3285 | 24.9634 |
| 2022 | 23.8231 | 23.5213 | 24.125 |
| 2023 | 22.9733 | 22.6772 | 23.2694 |

| Age Adjusted Rate (95% CI) WEST | | | |
| --- | --- | --- | --- |
| Year | Age Adjusted Rate | Age Adjusted Rate Lower 95% Confidence Interval | Age Adjusted Rate Upper 95% Confidence Interval |
| 1999 | 20.8411 | 20.3564 | 21.3258 |
| 2000 | 20.4241 | 19.9496 | 20.8986 |
| 2001 | 20.0621 | 19.5983 | 20.5258 |
| 2002 | 19.87 | 19.4134 | 20.3267 |
| 2003 | 20.6078 | 20.1484 | 21.0672 |
| 2004 | 20.1537 | 19.7036 | 20.6037 |
| 2005 | 20.8259 | 20.3754 | 21.2765 |
| 2006 | 20.9532 | 20.5073 | 21.3991 |
| 2007 | 20.4689 | 20.0339 | 20.9039 |
| 2008 | 20.4784 | 20.0491 | 20.9077 |
| 2009 | 19.671 | 19.2563 | 20.0857 |
| 2010 | 20.3626 | 19.9448 | 20.7804 |
| 2011 | 20.0098 | 19.6028 | 20.4169 |
| 2012 | 19.448 | 19.0522 | 19.8437 |
| 2013 | 19.1356 | 18.7483 | 19.5229 |
| 2014 | 18.5641 | 18.1876 | 18.9405 |
| 2015 | 18.8269 | 18.4542 | 19.1996 |
| 2016 | 18.5873 | 18.2212 | 18.9534 |
| 2017 | 18.5449 | 18.1825 | 18.9073 |
| 2018 | 17.749 | 17.4002 | 18.0978 |
| 2019 | 16.5475 | 16.2148 | 16.8803 |
| 2020 | 18.6024 | 18.2538 | 18.9511 |
| 2021 | 21.453 | 21.0694 | 21.8367 |
| 2022 | 21.4407 | 21.0702 | 21.8113 |
| 2023 | 20.4149 | 20.0539 | 20.7758 |
