# Supplementary material for: Trends and disparities in urinary tract infections-related mortality in the United States from 1999 to 2023: Insights from CDC WONDER
Source: Medicine (Baltimore). 2026 May 22;105(21):e49032. doi: 10.1097/MD.0000000000049032 (PMC13201035; doi:10.1097/MD.0000000000049032)
Supplement: Supplementary file 9 [file medi-105-e49032-s009.docx]

**Supplemental Table 9: Age-Adjusted Mortality Rates per 1000,000 in the United States, 1999 to 2023 by ten years age-group.**

| Ten-Year Age Groups | Year | Crude Rate | Crude Rate Lower 95% Confidence Interval | Crude Rate Upper 95% Confidence Interval |
| --- | --- | --- | --- | --- |
| 25-34 years | 1999 | 0.2987 | 0.2452 | 0.3521 |
| 25-34 years | 2000 | 0.2757 | 0.2242 | 0.3273 |
| 25-34 years | 2001 | 0.3091 | 0.2542 | 0.3639 |
| 25-34 years | 2002 | 0.305 | 0.2504 | 0.3595 |
| 25-34 years | 2003 | 0.3542 | 0.2953 | 0.4131 |
| 25-34 years | 2004 | 0.3005 | 0.2463 | 0.3547 |
| 25-34 years | 2005 | 0.3235 | 0.2672 | 0.3798 |
| 25-34 years | 2006 | 0.2741 | 0.2224 | 0.3258 |
| 25-34 years | 2007 | 0.3122 | 0.2573 | 0.3672 |
| 25-34 years | 2008 | 0.3507 | 0.2928 | 0.4086 |
| 25-34 years | 2009 | 0.3217 | 0.2666 | 0.3768 |
| 25-34 years | 2010 | 0.3969 | 0.336 | 0.4579 |
| 25-34 years | 2011 | 0.3254 | 0.2707 | 0.3801 |
| 25-34 years | 2012 | 0.3451 | 0.2891 | 0.4011 |
| 25-34 years | 2013 | 0.3361 | 0.2812 | 0.391 |
| 25-34 years | 2014 | 0.3516 | 0.2959 | 0.4073 |
| 25-34 years | 2015 | 0.3466 | 0.2917 | 0.4016 |
| 25-34 years | 2016 | 0.3738 | 0.3171 | 0.4305 |
| 25-34 years | 2017 | 0.4124 | 0.3533 | 0.4715 |
| 25-34 years | 2018 | 0.3633 | 0.308 | 0.4185 |
| 25-34 years | 2019 | 0.3657 | 0.3104 | 0.421 |
| 25-34 years | 2020 | 0.4906 | 0.4266 | 0.5545 |
| 25-34 years | 2021 | 0.5385 | 0.4711 | 0.606 |
| 25-34 years | 2022 | 0.5341 | 0.4669 | 0.6012 |
| 25-34 years | 2023 | 0.5204 | 0.4541 | 0.5866 |
| 35-44 years | 1999 | 0.8186 | 0.7351 | 0.9021 |
| 35-44 years | 2000 | 0.742 | 0.6625 | 0.8215 |
| 35-44 years | 2001 | 0.8102 | 0.7271 | 0.8933 |
| 35-44 years | 2002 | 0.8109 | 0.7274 | 0.8945 |
| 35-44 years | 2003 | 0.8108 | 0.7268 | 0.8948 |
| 35-44 years | 2004 | 0.8105 | 0.7262 | 0.8948 |
| 35-44 years | 2005 | 0.8689 | 0.7813 | 0.9564 |
| 35-44 years | 2006 | 0.8302 | 0.7443 | 0.9161 |
| 35-44 years | 2007 | 0.8856 | 0.7964 | 0.9748 |
| 35-44 years | 2008 | 0.7964 | 0.7112 | 0.8815 |
| 35-44 years | 2009 | 0.8292 | 0.7415 | 0.9168 |
| 35-44 years | 2010 | 0.9131 | 0.8206 | 1.0055 |
| 35-44 years | 2011 | 0.8787 | 0.7876 | 0.9699 |
| 35-44 years | 2012 | 0.8885 | 0.7967 | 0.9803 |
| 35-44 years | 2013 | 0.9023 | 0.8097 | 0.9949 |
| 35-44 years | 2014 | 0.8911 | 0.7991 | 0.983 |
| 35-44 years | 2015 | 0.882 | 0.7906 | 0.9734 |
| 35-44 years | 2016 | 0.892 | 0.8 | 0.984 |
| 35-44 years | 2017 | 0.9737 | 0.878 | 1.0694 |
| 35-44 years | 2018 | 0.9521 | 0.858 | 1.0462 |
| 35-44 years | 2019 | 0.9098 | 0.8182 | 1.0014 |
| 35-44 years | 2020 | 1.208 | 1.103 | 1.3129 |
| 35-44 years | 2021 | 1.4146 | 1.3027 | 1.5265 |
| 35-44 years | 2022 | 1.4601 | 1.3468 | 1.5734 |
| 35-44 years | 2023 | 1.2886 | 1.183 | 1.3942 |
| 45-54 years | 1999 | 2.1379 | 1.9881 | 2.2878 |
| 45-54 years | 2000 | 2.0702 | 1.9249 | 2.2155 |
| 45-54 years | 2001 | 2.0667 | 1.9247 | 2.2087 |
| 45-54 years | 2002 | 2.1504 | 2.0067 | 2.2941 |
| 45-54 years | 2003 | 2.2269 | 2.0821 | 2.3716 |
| 45-54 years | 2004 | 2.2172 | 2.0741 | 2.3602 |
| 45-54 years | 2005 | 2.372 | 2.2256 | 2.5184 |
| 45-54 years | 2006 | 2.4904 | 2.3417 | 2.6391 |
| 45-54 years | 2007 | 2.3873 | 2.2429 | 2.5318 |
| 45-54 years | 2008 | 2.3527 | 2.2101 | 2.4952 |
| 45-54 years | 2009 | 2.3113 | 2.1706 | 2.4519 |
| 45-54 years | 2010 | 2.3885 | 2.2457 | 2.5313 |
| 45-54 years | 2011 | 2.4352 | 2.2906 | 2.5799 |
| 45-54 years | 2012 | 2.4916 | 2.3446 | 2.6386 |
| 45-54 years | 2013 | 2.5361 | 2.3869 | 2.6853 |
| 45-54 years | 2014 | 2.5219 | 2.3726 | 2.6712 |
| 45-54 years | 2015 | 2.6535 | 2.4999 | 2.8071 |
| 45-54 years | 2016 | 2.7742 | 2.6164 | 2.9321 |
| 45-54 years | 2017 | 2.7044 | 2.5478 | 2.861 |
| 45-54 years | 2018 | 2.7479 | 2.5887 | 2.9071 |
| 45-54 years | 2019 | 2.6618 | 2.5036 | 2.8199 |
| 45-54 years | 2020 | 3.5252 | 3.3421 | 3.7084 |
| 45-54 years | 2021 | 3.9053 | 3.7133 | 4.0973 |
| 45-54 years | 2022 | 3.6877 | 3.5005 | 3.8749 |
| 45-54 years | 2023 | 3.3462 | 3.168 | 3.5244 |
| 55-64 years | 1999 | 6.0476 | 5.735 | 6.3602 |
| 55-64 years | 2000 | 6.0845 | 5.7742 | 6.3948 |
| 55-64 years | 2001 | 6.0545 | 5.7501 | 6.3589 |
| 55-64 years | 2002 | 6.1678 | 5.8699 | 6.4656 |
| 55-64 years | 2003 | 6.4265 | 6.1296 | 6.7234 |
| 55-64 years | 2004 | 6.074 | 5.7918 | 6.3562 |
| 55-64 years | 2005 | 6.5467 | 6.2602 | 6.8332 |
| 55-64 years | 2006 | 6.3827 | 6.1056 | 6.6598 |
| 55-64 years | 2007 | 6.5382 | 6.2628 | 6.8135 |
| 55-64 years | 2008 | 6.8449 | 6.5674 | 7.1223 |
| 55-64 years | 2009 | 6.5639 | 6.2971 | 6.8308 |
| 55-64 years | 2010 | 6.6168 | 6.3529 | 6.8808 |
| 55-64 years | 2011 | 7.1541 | 6.8854 | 7.4228 |
| 55-64 years | 2012 | 6.9947 | 6.7308 | 7.2586 |
| 55-64 years | 2013 | 7.2285 | 6.9628 | 7.4943 |
| 55-64 years | 2014 | 7.0588 | 6.7987 | 7.3189 |
| 55-64 years | 2015 | 7.7964 | 7.5257 | 8.0671 |
| 55-64 years | 2016 | 7.7081 | 7.4408 | 7.9753 |
| 55-64 years | 2017 | 8.0413 | 7.7701 | 8.3125 |
| 55-64 years | 2018 | 7.9981 | 7.7285 | 8.2677 |
| 55-64 years | 2019 | 7.9437 | 7.6756 | 8.2119 |
| 55-64 years | 2020 | 9.86 | 9.5611 | 10.1589 |
| 55-64 years | 2021 | 10.9128 | 10.5998 | 11.2257 |
| 55-64 years | 2022 | 10.6331 | 10.3216 | 10.9447 |
| 55-64 years | 2023 | 9.7791 | 9.4795 | 10.0787 |
| 65-74 years | 1999 | 23.8342 | 23.1291 | 24.5393 |
| 65-74 years | 2000 | 22.337 | 21.654 | 23.0201 |
| 65-74 years | 2001 | 23.8466 | 23.1407 | 24.5525 |
| 65-74 years | 2002 | 22.5684 | 21.8818 | 23.2551 |
| 65-74 years | 2003 | 22.8637 | 22.1747 | 23.5528 |
| 65-74 years | 2004 | 22.3275 | 21.6497 | 23.0054 |
| 65-74 years | 2005 | 23.7955 | 23.0997 | 24.4913 |
| 65-74 years | 2006 | 22.7308 | 22.0565 | 23.4051 |
| 65-74 years | 2007 | 22.773 | 22.1066 | 23.4395 |
| 65-74 years | 2008 | 22.545 | 21.8951 | 23.1949 |
| 65-74 years | 2009 | 21.5889 | 20.964 | 22.2139 |
| 65-74 years | 2010 | 22.6127 | 21.9802 | 23.2452 |
| 65-74 years | 2011 | 22.4627 | 21.8431 | 23.0822 |
| 65-74 years | 2012 | 22.3094 | 21.7117 | 22.9072 |
| 65-74 years | 2013 | 21.9655 | 21.3871 | 22.544 |
| 65-74 years | 2014 | 22.3727 | 21.8021 | 22.9433 |
| 65-74 years | 2015 | 23.3208 | 22.7505 | 23.891 |
| 65-74 years | 2016 | 23.9012 | 23.3349 | 24.4675 |
| 65-74 years | 2017 | 23.8652 | 23.3094 | 24.4209 |
| 65-74 years | 2018 | 23.4813 | 22.9374 | 24.0252 |
| 65-74 years | 2019 | 22.9676 | 22.4382 | 23.497 |
| 65-74 years | 2020 | 27.9206 | 27.3466 | 28.4947 |
| 65-74 years | 2021 | 31.0282 | 30.4332 | 31.6233 |
| 65-74 years | 2022 | 31.9192 | 31.3168 | 32.5216 |
| 65-74 years | 2023 | 29.217 | 28.6481 | 29.7859 |
| 75-84 years | 1999 | 102.152 | 100.3604 | 103.9437 |
| 75-84 years | 2000 | 101.0502 | 99.2781 | 102.8223 |
| 75-84 years | 2001 | 97.9464 | 96.2179 | 99.675 |
| 75-84 years | 2002 | 97.4237 | 95.7114 | 99.136 |
| 75-84 years | 2003 | 97.492 | 95.7879 | 99.1962 |
| 75-84 years | 2004 | 95.7051 | 94.0227 | 97.3875 |
| 75-84 years | 2005 | 102.2042 | 100.4713 | 103.9371 |
| 75-84 years | 2006 | 98.021 | 96.3253 | 99.7168 |
| 75-84 years | 2007 | 96.9708 | 95.2837 | 98.658 |
| 75-84 years | 2008 | 96.5043 | 94.8205 | 98.1881 |
| 75-84 years | 2009 | 90.096 | 88.4657 | 91.7263 |
| 75-84 years | 2010 | 92.136 | 90.4898 | 93.7822 |
| 75-84 years | 2011 | 91.543 | 89.9092 | 93.1768 |
| 75-84 years | 2012 | 91.0143 | 89.3913 | 92.6374 |
| 75-84 years | 2013 | 88.4913 | 86.9013 | 90.0813 |
| 75-84 years | 2014 | 87.8044 | 86.2343 | 89.3745 |
| 75-84 years | 2015 | 89.8789 | 88.3042 | 91.4537 |
| 75-84 years | 2016 | 87.5046 | 85.9678 | 89.0414 |
| 75-84 years | 2017 | 86.6077 | 85.1036 | 88.1118 |
| 75-84 years | 2018 | 83.3356 | 81.8936 | 84.7777 |
| 75-84 years | 2019 | 79.7314 | 78.3465 | 81.1163 |
| 75-84 years | 2020 | 92.7268 | 91.2554 | 94.1983 |
| 75-84 years | 2021 | 100.5796 | 99.0355 | 102.1237 |
| 75-84 years | 2022 | 101.7948 | 100.3008 | 103.2888 |
| 75-84 years | 2023 | 95.3338 | 93.9217 | 96.7458 |
| 85+ years | 1999 | 417.4512 | 411.2379 | 423.6646 |
| 85+ years | 2000 | 404.6856 | 398.6301 | 410.7412 |
| 85+ years | 2001 | 393.2295 | 387.311 | 399.1481 |
| 85+ years | 2002 | 398.0949 | 392.1783 | 404.0114 |
| 85+ years | 2003 | 386.5051 | 380.7393 | 392.271 |
| 85+ years | 2004 | 382.4559 | 376.7709 | 388.141 |
| 85+ years | 2005 | 406.4092 | 400.6416 | 412.1769 |
| 85+ years | 2006 | 386.9559 | 381.4287 | 392.4831 |
| 85+ years | 2007 | 381.5821 | 376.1888 | 386.9754 |
| 85+ years | 2008 | 377.3788 | 372.0966 | 382.6611 |
| 85+ years | 2009 | 359.3426 | 354.2712 | 364.4141 |
| 85+ years | 2010 | 369.1134 | 364.0329 | 374.194 |
| 85+ years | 2011 | 367.3063 | 362.347 | 372.2656 |
| 85+ years | 2012 | 358.9403 | 354.1007 | 363.7799 |
| 85+ years | 2013 | 339.7073 | 335.0593 | 344.3552 |
| 85+ years | 2014 | 333.0125 | 328.4562 | 337.5689 |
| 85+ years | 2015 | 339.7718 | 335.2154 | 344.3282 |
| 85+ years | 2016 | 333.8071 | 329.324 | 338.2903 |
| 85+ years | 2017 | 327.872 | 323.4594 | 332.2847 |
| 85+ years | 2018 | 311.8189 | 307.5407 | 316.0972 |
| 85+ years | 2019 | 300.5318 | 296.3509 | 304.7127 |
| 85+ years | 2020 | 339.7803 | 335.3527 | 344.2079 |
| 85+ years | 2021 | 368.8906 | 364.0208 | 373.7603 |
| 85+ years | 2022 | 352.0886 | 347.5219 | 356.6552 |
| 85+ years | 2023 | 353.0601 | 348.381 | 357.7391 |
